# Castleman disease of stomach treated by endoscopic submucosal dissection: a case report and literature review

**DOI:** 10.3389/fonc.2025.1563545

**Published:** 2025-04-04

**Authors:** Ting-Ting Sun, Xue-Guo Sun, Ti-Dong Shan, Peng Zhao, Yan-Yan Lu, Qian Li, Fu-Guo Liu

**Affiliations:** ^1^ Department of Gastroenterology, The Affiliated Hospital of Qingdao University, Qingdao, Shandong, China; ^2^ Gastroenterology, Qingdao Medical College of Qingdao University, Qingdao, Shandong, China; ^3^ Department of Pathology, The Affiliated Hospital of Qingdao University, Qingdao, Shandong, China

**Keywords:** unicentric Castleman disease, gastrointestinal tract, Castleman disease, endoscopic submucosal dissection, histopathology, case report

## Abstract

**Background:**

Castleman disease (CD) is a relatively rare benign lymphoproliferative disorder of the lymphoid tissue. According to clinical manifestations, it is classified into two types: unicentric CD (UCD) and multicentric CD (MCD). Pathological subtypes include hyaline-vascular (HV), plasma cell (PC), and mixed (MV). Gastrointestinal CD is extremely rare, and limited information is available regarding its clinical presentation and management.

**Case Summary:**

We report a case of a patient who presented with paroxysmal epigastric pain for 4 years. Laboratory tests showed no remarkable abnormalities, whereas CT revealed endogenous occupancy on the side of the greater curvature of the stomach. Ultrasonographic endoscopy demonstrated hypoechoic, well-defined foci. The lesion initially suspected to be an inflammatory fibroma was subsequently pathologically confirmed as HV-UCD following endoscopic submucosal dissection. The lesion was completely resected, and the patient showed no signs of recurrence during 7 months of follow-up.

**Conclusion:**

Gastrointestinal CD is rare and should be differentiated from other occupying lesions. Its definitive diagnosis relies on histopathology.

## Introduction

Castleman disease (CD) is a rare, benign lymphoproliferative disorder of unknown etiology and pathogenesis, clinically characterized by significant painless lymph node enlargement (1).CD encompasses at least four heterogeneous subtypes with differences in etiology, symptom presentation, treatment and outcomes (2). The disease is classified into two main categories: unicentric CD (UCD) and multicentric CD (MCD). MCD is further divided into three types based on the underlying drivers: HHV8-associated MCD (HHV8-MCD), POEMS-associated MCD (POEMS-MCD), and idiopathic MCD (iMCD) (3).Patients with MCD typically exhibit systemic multi-organ involvement and an inflammatory response, which may or may not be accompanied by abnormal laboratory findings (4).UCD is often asymptomatic or may cause discomfort due to compression of surrounding tissues from the enlarged lymph nodes. UCD is most often seen in the mediastinum, abdominal cavity, and other areas with lymph nodes (5). Occurrence in the lumen of the gastrointestinal tract is extremely rare. Currently, there is no standardized treatment protocol for CD.

We report a case of gastric CD successfully treated with endoscopic submucosal dissection (ESD), with the diagnosis pathologically confirmed. To the best of our knowledge, this represents the first reported case of a lesion originating in the submucosa and managed with endoscopic resection. Furthermore, we review relevant literature to improve understanding of gastrointestinal CD.

## Case presentation

**Figure 1:**
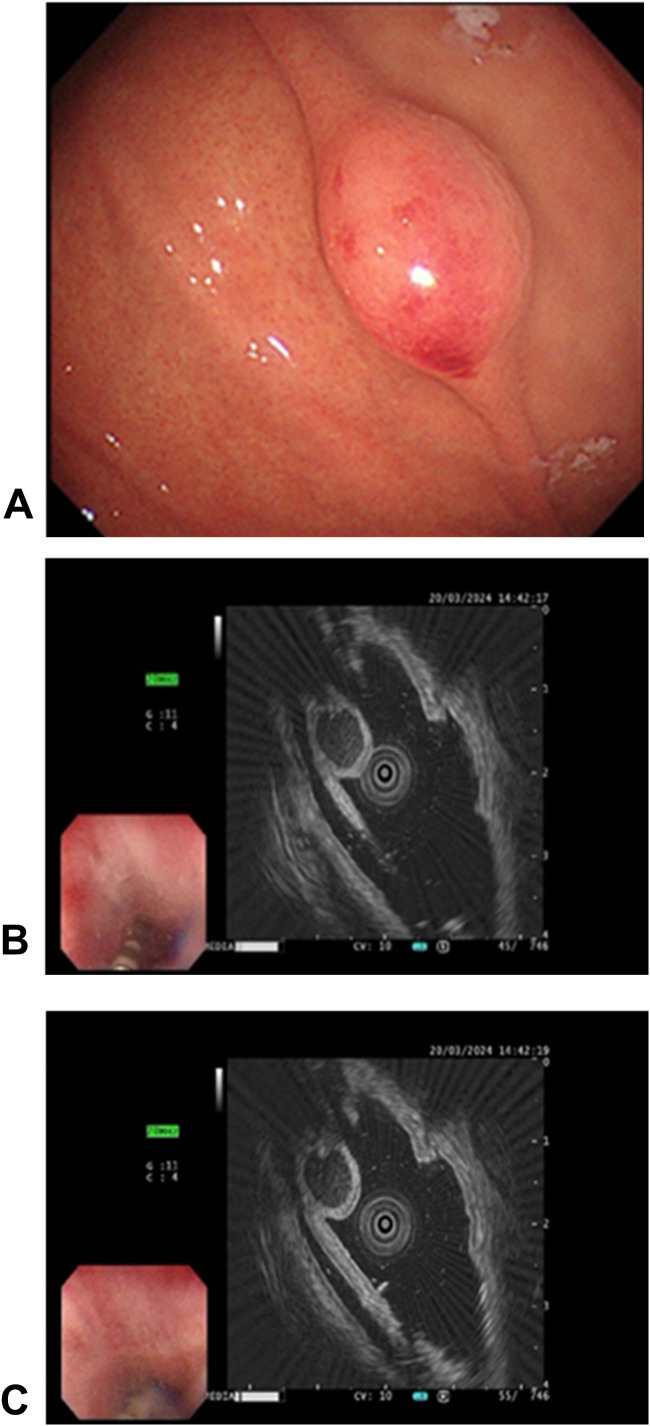
Endoscopic manifestations of the mass. **(A)** The gastric body exhibited a spherical, nontender submucosal elevation classified as type 0-Is according to the Paris classification of superficial gastrointestinal tumor lesions (0-Is: flattened, elevated type). The surface appears congested and reddish, indicating mucosal changes; **(B, C)** Endoscopic ultrasonography of the lesion. A mixed hypoechoic mass, characterized by inhomogeneous internal echogenicity, visible tubular structure, and posterior attenuation, is observed. The hemispherical mass extends into the cavity and originates from the submucosa.

A 45-year-old woman was admitted to our hospital with a 4-year history of paroxysmal pain in the epigastric region, and strongly requested an endoscopic examination. Before hospitalization, an electronic fiberoptic gastroduodenoscopy revealed a submucosal bulge, measuring approximately 8 mm x 12 mm, located on the greater curvature side of the upper part of the gastric body. The lesion was 0-Is type in size, with a congested and blood-filled surface (**Figure 1A** **).** Subsequent endoscopic ultrasound revealed a mixed hypoechoic mass with heterogeneous internal echogenicity, a tubular structure, posterior attenuation, and a hemispherical shape protruding into the lumen, originating from the submucosal layer, suspected to be inflammatory fibroids (**Figures 1B, C** **).** One month later, the patient was admitted for further evaluation and treatment.

**Figure 2:**
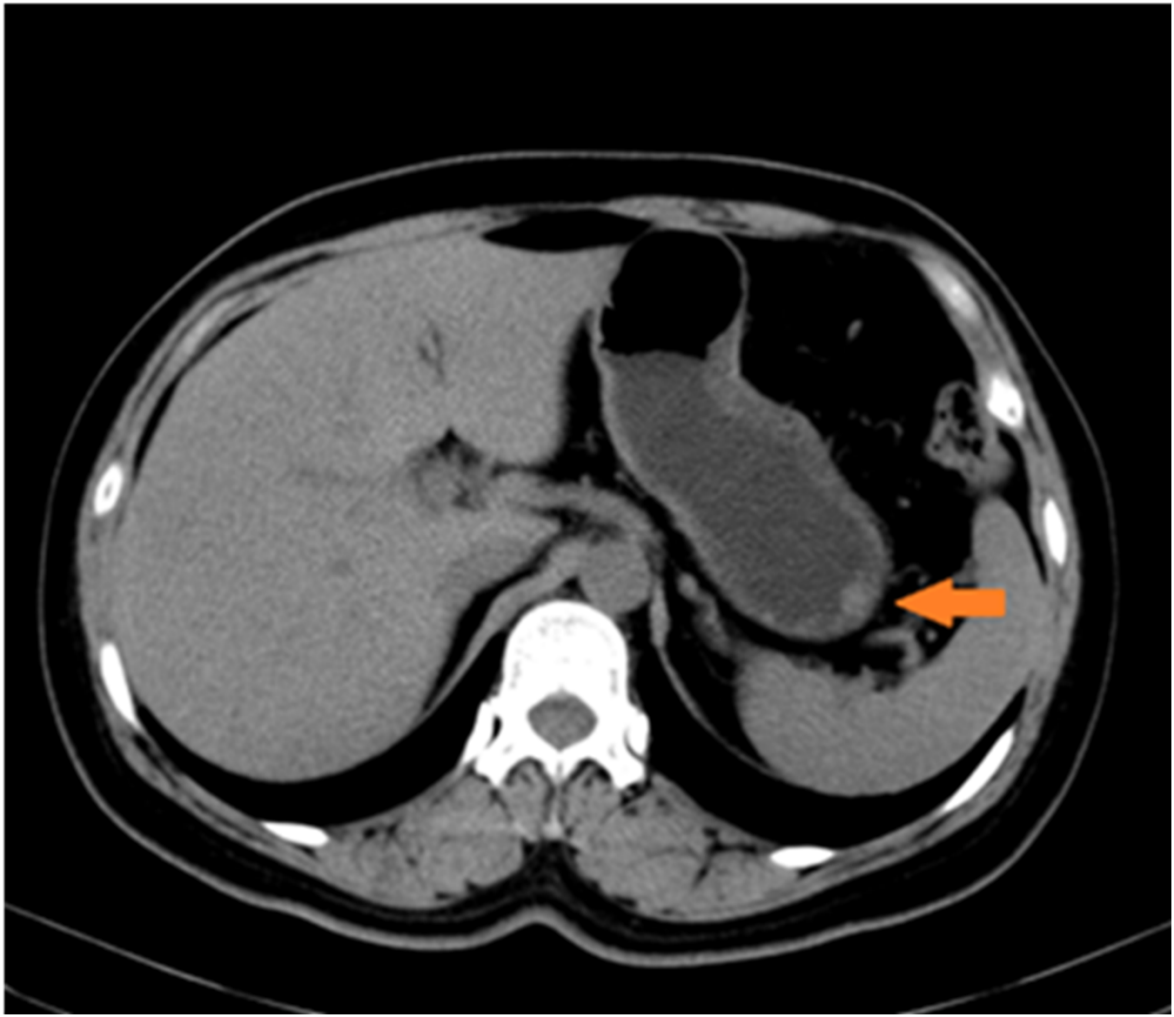
Radiological findings on the greater curvature of the stomach. Slight thickening of the lateral wall of the greater curvature of the gastric body is observed, presenting as a rounded soft-tissue density shadow.

Upon admission, no superficial lymph node enlargement was noted throughout the body, and cardiac, pulmonary, or abdominal examinations showed no abnormal signs. The patient’s past medical, personal, and family histories were unremarkable. Laboratory tests were performed, including blood count, liver and kidney function, electrolytes, carcinoembryonic antigen, cancer antigen 19-9, or alpha fetoprotein. Due to technical limitations, HHV-8 testing cannot be performed. Epigastric CT revealed slight thickening of the lateral wall of the greater curvature and a poorly defined, rounded soft tissue density shadow projecting into the gastric cavity. (**Figure 2**). Chest CT revealed no evidence of enlarged lymph nodes or pulmonary lesions. Based on the combined findings from the physical examination and chest and abdominal CT imaging, other sites of involvement were preliminarily excluded.

**Figure 3:**
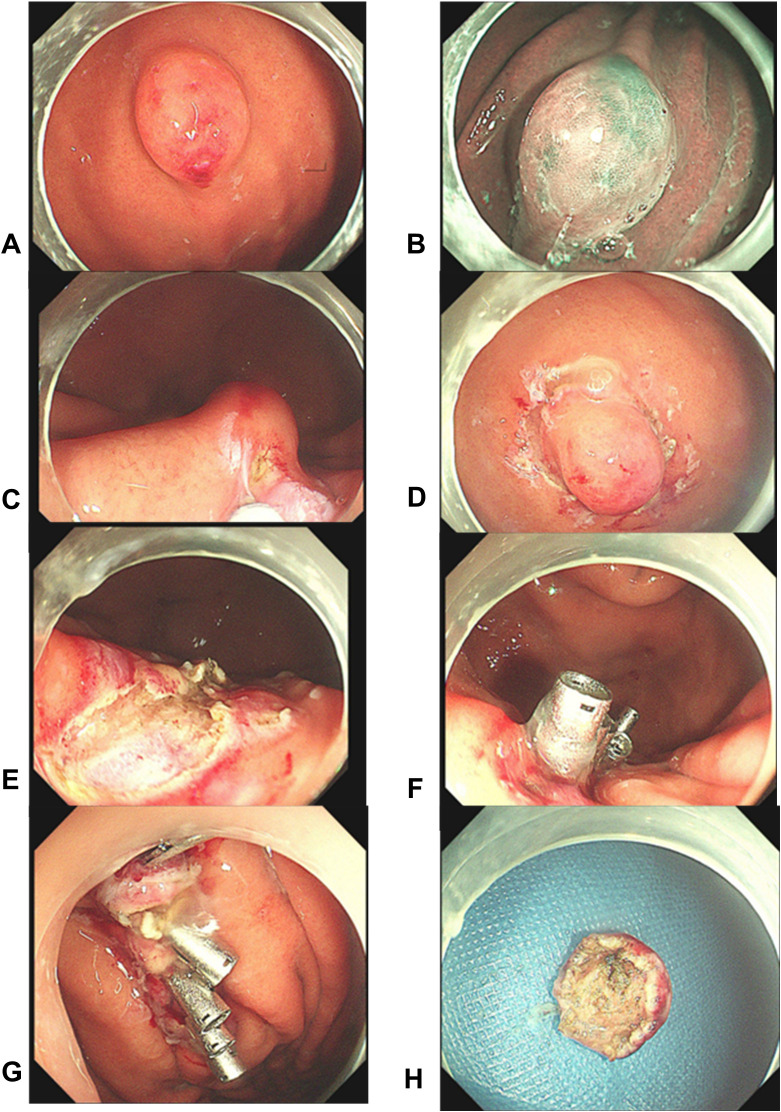
Endoscopic findings and post-resection observations. **(A–D)** The mass was observed using different endoscopic modalities. Subsequently, the margins were traced, and a submucosal dissection was performed near the muscular surface; **(E–H)** Post-resection views show the intact muscular layer at the site, confirming complete excision without residual trauma. Five titanium clips were then used to close the wound. The mass was completely excised with clear margins and the wound remained intact.

**Figure 4:**
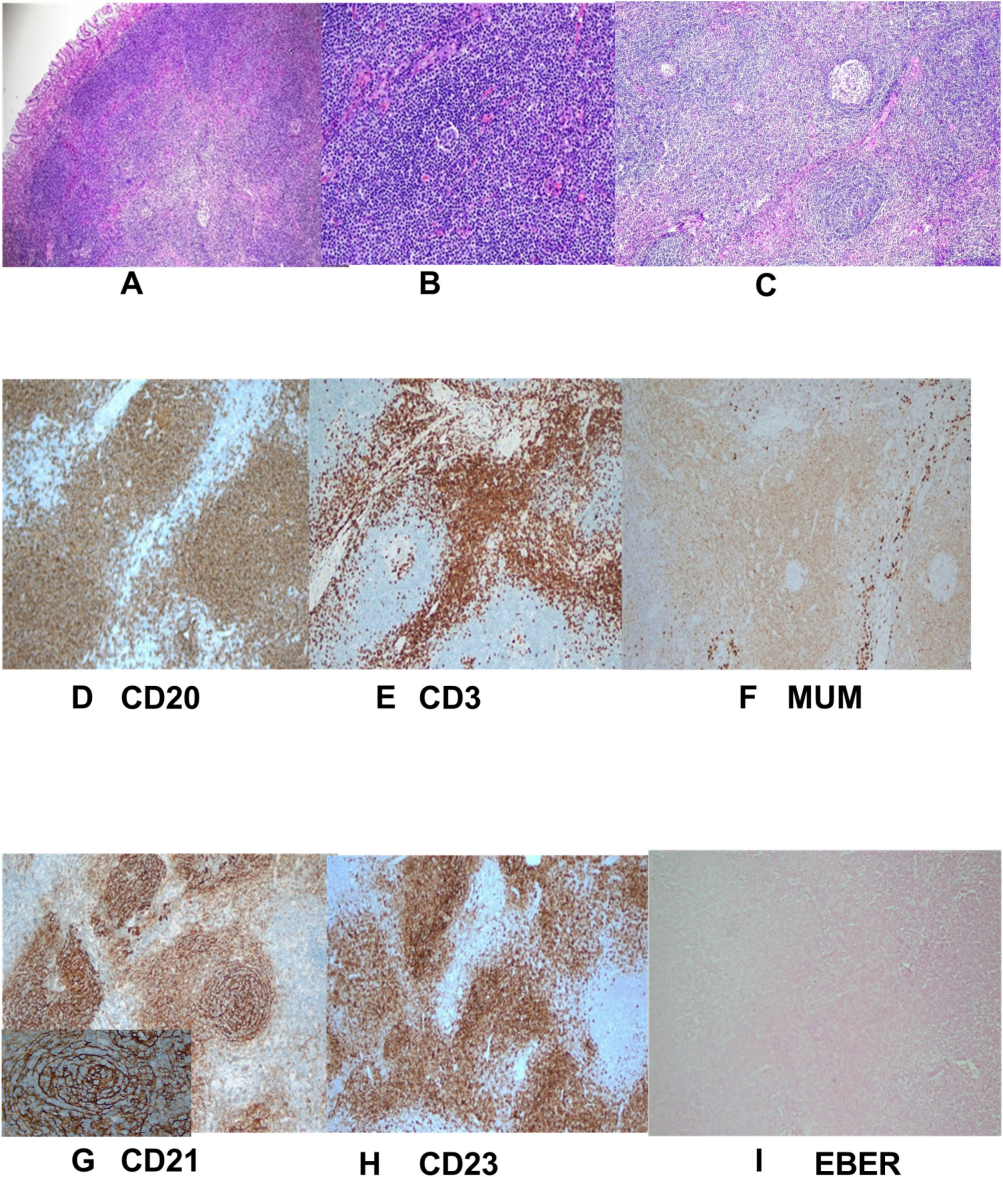
Pathological and immunohistochemical manifestations. **(A–C)** Postoperative histopathology (H&E staining) revealed significant hyperplasia of lymphoid tissue in the submucosal tissue of the gastric body. The tissue primarily composed of small lymphocytes with some areas of uniform cell size and atrophic follicular centers. No *Hp* was detected; **(D–I)** Immunohistochemistry showed numerous small blood vessels, some of which were hyalinized. Exhibiting changes consistent with Castleman disease. Specific findings included CD20 markers (B cells +), CD3 markers (T cells +), MUM1 (plasma cells +), CD23 markers (condylomata cells +), CD21 markers (follicular dendritic network +), EBER *in situ* hybridization was a negative with a positive control.

The lesion was excised using standard ESD. Intraoperatively, narrow band imaging showed a fundic glandular structure with regular microvascular patterns on the surface mucosa. The ESD procedure was performed as follows: (1) marking the lesion with a 5-mm margin, (2) submucosal injection of indigo carmine saline solution, and (3) circumferential dissection using a FlushKnife. The lesion was found to originate from the submucosal layer without invading the lamina propria (**Figure 3**). Submucosal dissection was carefully conducted just above the muscularis propria layer, and hemostasis was achieved using electrocoagulation forceps to gently coagulate the exposed vessels. Finally, tissue clips were applied to close the wound, minimizing the risk of delayed hemorrhage and perforation. Postoperative histopathological examination revealed small lymphocytic hyperplasia accompanied by atrophic follicular centers within the submucosal layer of the gastric body. Immunohistochemical analysis revealed positivity for CD20 (B cells+), CD3 (T cells+), MUM1 (plasma cells+), CD23 (follicular dendritic cells+), and CD21 (follicular dendritic network+) (**Figure 4**), supporting a diagnosis of gastric CD. The patient was administered a proton pump inhibitor preparation after ESD, which improved epigastric pain to a certain extent. And the postoperative follow-up visit, abdominal pain had resolved, leading to the hypothesis that abdominal pain was caused by the intra-gastric mass or concomitant chronic gastritis.

## Discussion

CD is a chronic lymphoproliferative disorder first reported in 1954 (1). The Castleman Disease Collaborative Network classifies CD into two main categories: UCD and MCD (3). Histopathologically, CD can be classified as hyaline vascular (HV), plasma cell (PC) and mixed (MV) types. HV-CD is characterized by atrophic germinal centers and vascular hyperplasia with vitreous degeneration, while PC-CD features hyperplastic germinal centers and diffuse polymorphic PC infiltration. The MV type exhibits features of both HV and PC types, presenting with atrophic follicles along with extensive PC infiltration (6, 7).

Epidemiological studies indicate CD can occur across all age groups, with adults (≥18 years) accounting for the majority of case. The median age of onset ranges between 20 and 50 years, with no significant sex differences in prevalence (1). The most common sites of Castleman’s disease include the mediastinum (63%), abdominal cavity (11%), and axillary region (4%) (8). According to current literature, abdominal Castleman’s disease can manifest in various locations, including the pancreas, peripancreatic region, retroperitoneum (9)、hepatogastric hiatus (10)、mesentery (11)、gastrointestinal tract, and lymph nodes within the abdominal cavity. The incidence of pancreas and peripancreas and retroperitoneum is about 7% (12), and there are no clear statistics on the incidence of the remaining sites.

Current research suggests that MCD is mostly associated with HHV-8 infection, aberrant interleukin (IL)-6 expression, and related malignancies (13). MCD is more likely to occur in individuals with chronic HIV infection and in older or immunocompromised patients who are HIV-negative (14, 15). Unlike UCD, patients with MCD often present with systemic inflammatory and autoimmune reactions. Among the common pathological subtypes of MCD, the PC type is the most prevalent (2), and is associated with a higher risk of developing complications (1).

UCD is a clonally proliferative tumor originating from mesenchymal cells, particularly follicular dendritic cells (16). Lymph node enlargement in UCD is significantly more pronounced than that observed in MCD (5). Common sites of UCD involvement include lymph node regions, such as the chest, neck, abdomen, and retroperitoneum (17). HV-UCD is common in UCD. The prognosis of patients with UCD is generally good; however, cases combined with paraneoplastic aspergillosis and occlusive bronchiectasis have poorer outcomes (18).

The incidence of Castleman’s disease localized to the abdominal region is approximately 7%, while cases involving the gastrointestinal tract lumen are exceptionally rare. Reports of CD occurring in the stomach are rare, with only 15 cases of CD in the gastrointestinal tract reported. Feng (19) described a young male with a lateral exophytic mass in the stomach presenting with epigastric discomfort. The patient underwent laparoscopic gastric tumor resection, and postoperative pathological analysis suggested an HV-type UCD. Palvia (20) reported a case of a young woman with iMCD, presenting with an endogenous mass in the gastric fundus. This case was associated with HHV-6 infection, antiphospholipid antibody syndrome, and splenic infarction. The patient achieved symptomatic relief following treatment with siltuximab and tocilizumab and currently remains under follow-up. Wang et al. (21) reported a case of a patient with recurrent epigastric discomfort. Gastroscopy revealed a subepithelial mass in the lumen of the stomach with intact mucosa. Ultrasonographic endoscopy showed a homogeneous, hypoechoic elliptical lesion originating from the muscularis propria, and pathological examination confirmed the diagnosis of MV-UCD following endoscopic resection. Shariati (9) reported another case involving a round, well-demarcatedcalcified masswithin the lamina propria. Neuroendocrine tumor could not be excluded based on clinical manifestations and laboratory tests. A Cesarean section was performed, and a HV-UCD with a rich blood supply was found. Wengrower (22) presented a case series study involving two patients with MCD who presented to the clinic with abdominal pain. Endoscopic findings showed umbilical lesions and multiple erosions in the gastric sinuses. Biopsy results indicated non-specific inflammation, potentially representing primary disease symptoms. Kartal (23) showed another case occuring in the terminal ileum and ileocecal valve, leading to complete intestinal obstruction. Abdominal CT revealed thickening of the terminal ileum wall and mesenteric lymph node enlargement. Postoperative pathology indicated HV-type UCD. Lai et al (24) and Korukluoglu (25) showed two cases of duodenal HV-type UCD characterized by homogeneous soft-tissue density on enhanced CT. Both cases were treated with surgical resection. Additionally, Hata (26) showed a case of rectal PC-UCD combined with gastric adenocarcinoma. The lesion presented endoscopically as a bleeding polypoid mass with a short and thick tip, occupying approximately half of the intestinal lumen. No recurrence was observed at 22 months post-surgery. Moss (27) indicated that jejunal lymphadenopathy secondary to HV-MCD was associated with systemic POEMS syndrome and myocardial infarction. van Rhee et al. (28) reported four cases of multicentric Castleman disease (MCD) primarily involving the intestinal tract and demonstrated a strong correlation between MCD and pathological findings from associated lymph node biopsies. Treatment was tailored to each patient’s clinical condition, incorporating surgical intervention and pharmacologic therapy as needed.

Our findings indicate that gastrointestinal CD most commonly occurs in the stomach, with UCD being the predominant form, typically without an obvious systemic inflammatory reaction. UCD typically presents as a solitary, mucosa-covered exophytic mass within the gastric lumen. While previously reported cases originated in the lamina propria, the current case arose from the submucosa. Ultrasonography typically reveals a homogeneous, hypoechoic mass, occasionally with calcifications. MCD often involves multiple gastrointestinal sites, manifesting as diffuse erosions, ulcers, and nonspecific inflammation in the stomach or concurrent involvement of multiple small bowel segments. MCD is frequently associated with systemic inflammatory responses and abnormal laboratory findings. Differentiating CD of the gastrointestinal tract from other submucosal masses—such as lymphomas, follicular dendritic cell tumors, inflammatory fibromas, and neuroendocrine tumors—can be challenging (29–31). Studies have shown that isolated hypoechoic, homogeneous, well-demarcated masses with prominent echogenic features of the vascular system, increased elasticity, and uniformly enhanced CT manifestations are highly indicative of UCD (32, 33), The presence of calcifications or necrotic foci, particularly in larger masses, may aid in distinguishing Castleman disease from lymphoma (34), In addition to magnetic resonance (MR) imaging and ultrasonography, positron emission tomography-computed tomography (PET-CT) can also aid in diagnosis, with PET-CT being considered a superior option (5, 35). Histopathological examination remains essential for definitive diagnosis due to the nonspecific nature of clinical presentations (36).

Based on the aforementioned cases, patients presenting with UCD and mild systemic inflammatory responses may be considered for surgical resection, including both open and laparoscopic approaches (1). Asymptomatic patients can be monitored, whereas patients with pressure symptoms may be treated with rituximab combined with glucocorticoids or chemotherapy. Surgical resection is optional after evaluation of lesion shrinkage. If lesions are associated with systemic hyperinflammation and difficult to resect, treatment with IL-6 monoclonal antibody (siltuximab) can be combinate with glucocorticoids or thalidomide-cyclophosphamide- prednisone (TCP) therapy (37, 38). Radiotherapy, embolization, and neoadjuvant therapy are additional treatment options (39). The classification of MCD is more complex, with rituximab-based regimens being the preferred choice for HHV-8 MCD, For non-heavy MCD, situximab is preferred, with TCP and rituximab-based regimens as alternative options. For patients with severe MCD, the recommended first-line treatment is a combination of siltuximab and high-dose glucocorticoids (36). For patients with UCD or MCD who develop intestinal obstruction secondary to lymphoid tissue hyperplasia or mass compression, surgical resection remains a viable treatment option.

In our case, the characteristics of the mass detected by endoscopy and ultrasonogastroscopic findings suggested that the lesion originated in the submucosal layer and was well demarcated from the lamina layer. Subsequently, a comprehensive assessment of the possibility of complete endoscopic resection and pathologic examination was performed. Therefore, the diagnosis of EUS-FNA/B was not made. Among the available endoscopic resection modalities, endoscopic mucosal resection (EMR) and ESD were considered. However, EMR often poses challenges in achieving complete removal of the submucosal layer, increasing the risk of incomplete mass resection. In contrast, ESD allows for en bloc of the submucosal layer while preserving the mucosal layer above the intrinsic muscularis layer. Pathological examination confirmed the final and definitive diagnosis, suggesting the effectiveness of ESD as a treatment modality for intraluminal gastrointestinal CD.

## Conclusion

Gastric CD is extremely rare, with only a few reported. In our case, the UCD was not associated with systemic inflammatory reactions and presented as an endogenous growth with a reddish surface on the greater curvature of the stomach. Ultrasonographic endoscopy revealed that the lesion originated from the submucosal layer and was completely resected using ESD. This method proved to be a safe and effective approach for resecting CD with intraluminal growth in the gastrointestinal tract. Before local excision to target the lesion, distinguishing CD from other gastric tumors such as follicular dendritic cell tumor, calcified fibromas, and inflammatory fibromas is essential. Comprehensive evaluation, including relevant hematological test, imaging, and endoscopic ultrasound, should be performed to identify any coexisting autoimmune disease or inflammatory states, ensuring early identification and intervention.

## Data Availability

The original contributions presented in the study are included in the article/supplementary material. Further inquiries can be directed to the corresponding author.

## References

[1] Zhang L Dong YJ Peng HL Li H Zhang MZ Wang HH. A national, multicenter, retrospective study of Castleman disease in China implementing CDCN criteria. Lancet Reg Health West Pac. (2023) 34:100720. doi: 10.1016/j.lanwpc.2023.100720 37283978 PMC10240357

[2] DispenzieriAFajgenbaumDC. Overview of castleman disease. Blood. (2020) 135:1353–64. doi: 10.1182/blood.2019000931 32106302

[3] Fajgenbaum DC Uldrick TS Bagg A Frank D Wu D Srkalovic G. International, evidence-based consensus diagnostic criteria for HHV-8-negative/idiopathic multicentric Castleman disease. Blood. (2017) 129:1646–57. doi: 10.1182/blood-2016-10-746933 PMC5364342 28087540

[4] Polizzotto MN Uldrick TS Wang V Aleman K Wyvill KM Marshall V. Human and viral interleukin-6 and other cytokines in Kaposi sarcoma herpesvirus-associated multicentric Castleman disease. Blood. (2013) 122:4189–98. doi: 10.1182/blood-2013-08-519959 PMC3868925 24174627

[5] Wong RSM. Unicentric castleman disease. Hematol Oncol Clin North Am. (2018) 32:65–73. doi: 10.1016/j.hoc.2017.09.006 29157620

[6] Keller AR Hochholzer L Castleman B. Hyaline-vascular and plasma-cell types of giant lymph node hyperplasia of the mediastinum and other locations. Cancer. (1972) 29:670–83. doi: 10.1002/1097-0142(197203)29:3<670::AID-CNCR2820290321>3.0.CO;2-# 4551306

[7] Herrada J Cabanillas F Rice L Manning J Pugh W. The clinical behavior of localized and multicentric Castleman disease. Ann Intern Med. (1998) 128:657–62. doi: 10.7326/0003-4819-128-8-199804150-00010 9537940

[8] Bonekamp D Horton KM Hruban RH Fishman EK. Castleman disease: the great mimic. Radiographics. (2011) 31:1793–807. doi: 10.1148/rg.316115502 21997995

[9] Shariati F Verter E Chang W Huang L Joshi V. Castleman disease presenting as an abdominal mass. ACG Case Rep J. (2017) 4:e71. doi: 10.14309/crj.2017.71 28584844 PMC5449581

[10] Xu XY Liu XQ Du HW Liu JH. Castleman disease in the hepatic-gastric. space: A Case Rep World J Clin cases. (2019) 7:4391–7. doi: 10.12998/wjcc.v7.i24.4391 PMC6940338 31911923

[11] Kim SH Min BW Kim WB Park SS Um JW Lee JB. Mesenteric castleman’s disease. Yonsei Med J. (2005) 46:289–91. doi: 10.3349/ymj.2005.46.2.289 PMC2823027 15861504

[12] Takihara H Yamakawa G Baba Y Takahashi M Ishihara T. Castleman disease. Unusual retroperitoneal location indistinguishable from Malignant tumor in preoperative angiographic appearance. Urology. (1993) 41:162–4. doi: 10.1016/0090-4295(93)90173-8 8497992

[13] Peterson BA Frizzera G. Multicentric castleman’s disease. Semin Oncol. (1993) 20:636–47. 8296200

[14] Collins LS Fowler A Tong CY de Ruiter A. Multicentric Castleman’s disease in HIV infection. Int J STD AIDS. (2006) 17:19–24. doi: 10.1258/095646206775220496 16409673

[15] Műzes G Sipos F Csomor J Sréter L. Multicentric Castleman’s disease: a challenging diagnosis. Pathol Oncol Res. (2013) 19:345–51. doi: 10.1007/s12253-013-9619-z 23516126

[16] Dossier A Meignin V Fieschi C Boutboul D Oksenhendler E Galicier L. Human herpesvirus 8-related Castleman disease in the absence of HIV infection. Clin Infect Dis. (2013) 56:833–42. doi: 10.1093/cid/cis1009 23223599

[17] Wu J Lu AD Zhang LP Zuo YX Jia YP. Study of clinical outcome and prognosis in pediatric core binding factor-acute myeloid leukemia. Zhonghua Xue Ye Xue Za Zhi. (2019) 40:52–7. doi: 10.3760/cma.j.issn.0253-2727.2019.01.010 (Article in Chinese) PMC7351698 30704229

[18] Fujimoto W Kanehiro A Kuwamoto-Hara K Saitoh M Nakakita T Amagai M. Paraneoplastic pemphigus associated with Castleman’s disease and asymptomatic bronchiolitis obliterans. Eur J Dermatol. (2002) 12:355–9. 12095881

[19] Feng ZQ Sheng CR Chen WC. Gastric Castleman’s disease: A case report and literature review. J Gastroenterol. (2023) 28:62–4. (Article in Chinese)

[20] Palvia AR Saha P Nandi AR Damera AR Suresh A. Unraveling the complexities of idiopathic multicentric castleman disease and its multi-systemic associations: A case report. Cureus. (2024) 16:e64935. doi: 10.7759/cureus.64935 39161530 PMC11332968

[21] Wang J Wang B Chen DF. A rare submucosal tumor of the stomach. Gastroenterology. (2013) 144:e5–6. doi: 10.1053/j.gastro.2012.11.028 23462130

[22] Wengrower D Libson E Okon E Goldin E. Gastrointestinal manifestations in Castleman’s disease. Am J Gastroenterol. (1990) 85:1179–81. 2389730

[23] Kartal A Atlı E Vural G Ferhatoğlu MF Filiz A. Castleman’s disease presenting with mechanical intestinal obstruction: A rare case. Ulus Travma Acil Cerrahi Derg. (2020) 26:144–7. doi: 10.5505/tjtes.2018.42273 31942736

[24] Lai S Hu C Zheng Q. Uncommon presentation of Castleman disease in the duodenum: a case description and computed tomography imaging analysis. Quant Imaging Med Surg. (2024) 14:7749–52. doi: 10.21037/qims-24-704 PMC11485329 39429597

[25] Korukluoglu B Ergul E Yalcin S Mehmet Ozgun Y Kusdemir A. Castleman’s disease of the duodenum: a case report. Acta Chir Belg. (2009) 109:240–1. doi: 10.1080/00015458.2009.11680414 19499690

[26] Hata T Ikeda M Ikenaga M Yasui M Shingai T Yamamoto H. Castleman’s disease of the rectum: report of a case. Dis Colon Rectum. (2007) 50:389–94. doi: 10.1007/s10350-006-0783-z 17171476

[27] Moss SF Thomas DM Mulnier C McGill IG Hodgson HJ. Intestinal lymphangiectasia associated with angiofollicular lymph node hyperplasia (Castleman’s disease). Gut. (1992) 33:135–7. doi: 10.1136/gut.33.1.135 PMC1373880 1740268

[28] Patra S Acharya P Padhi S Chandra Samal S Mishra P Panigrahi MK. Signature of Castleman disease in gastrointestinal mucosal biopsies and resected sp*ecimen: report of four cases and literature review* . Pathology. (2023) 55:703–7. doi: 10.1016/j.pathol.2022.11.011 36907779

[29] Hill AJ Tirumani SH Rosenthal MH Shinagare AB Carrasco RD Munshi NC. Multimodality imaging and clinical features in Castleman disease: single institute experience in 30 patients. Br J Radiol. (2015) 88:20140670. doi: 10.1259/bjr.20140670 25710283 PMC4628472

[30] Marbaniang E Khonglah Y Dey B Shunyu B Gogoi B. Castleman’s disease associated with calcifying fibrous tumor: A rare association with review of literature. J Lab Physicians. (2019) 11:171–3. doi: 10.4103/JLP.JLP_16_19 PMC6543940 31160859

[31] Agaimy A Wünsch PH. Follicular dendritic cell tumor of the gastrointestinal tract: Report of a rare neoplasm and literature review. Pathol Res Pract. (2006) 202:541–8. doi: 10.1016/j.prp.2006.01.013 16564140

[32] Ko SF Hsieh MJ Ng SH Lin JW Wan YL Lee TY. Imaging sp*ectrum of Castleman’s disease* . AJR Am J Roentgenol. (2004) 182:769–75. doi: 10.2214/ajr.182.3.1820769 14975984

[33] Panagiotakopoulos D Mouchtouris A Zarakostas M Rontogianni D Athanasiadou P. Endosonographic features of unicentric Castleman Disease. Endosc Ultrasound. (2014) 3:S10–1. doi: 10.4103/2303-9027.129513 PMC4569904 26425506

[34] Kim TJ Han JK Kim YH Kim TK Choi BI. Castleman disease of the abdomen: imaging sp*ectrum and clinicopathologic correlations* . J Comput Assist Tomogr. (2001) 25:207–14. doi: 10.1097/00004728-200103000-00008 11242214

[35] Lee ES Paeng JC Park CM Chang W Lee WW Kang KW. Metabolic characteristics of Castleman disease on 18F-FDG PET in relation to clinical implication. Clin Nucl Med. (2013) 38:339–42. doi: 10.1097/RLU.0b013e3182816730 23429387

[36] Lymphocyte Disease Group of the Hematology Branch of the Chinese Medical Association Hemato-Oncology Committee of the Chinese Anti-Cancer Association Chinese Castleman’s Disease Collaborative Group. Expert consensus on diagnosis and treatment of castleman’s disease in China (2021 edition). Chin J Haematology. (2021) 42(7):529–34. doi: 10.3760/cma.j.issn.0253-2727.2021.07.001

[37] van Rhee F Voorhees P Dispenzieri A Fosså A Srkalovic G Ide M. International, evidence-based consensus treatment guidelines for idiopathic multicentric Castleman disease. Blood. (2018) 132:2115–24. doi: 10.1182/blood-2018-07-862334 PMC6238190 30181172

[38] Zhang L Zhao AL Duan MH Li ZY Cao XX Feng J. Phase 2 study using oral thalidomide-cyclophosphamide-prednisone for idiopathic multicentric Castleman disease. Blood. (2019) 133:1720–8. doi: 10.1182/blood-2018-11-884577 30760451

[39] Mohan M Meek JC Meek ME Broadwater R Alapat D van Rhee F. Combinatorial treatment for unresectable unicentric Castleman disease. Eur J Haematol. (2021) 107:484–8. doi: 10.1111/ejh.v107.4 34242421

